# The Relationship between P3 Amplitude and Working Memory Performance Differs in Young and Older Adults

**DOI:** 10.1371/journal.pone.0063701

**Published:** 2013-05-07

**Authors:** Emi Saliasi, Linda Geerligs, Monicque M. Lorist, Natasha M. Maurits

**Affiliations:** 1 Department of Neurology, University Medical Center Groningen, University of Groningen, Groningen, The Netherlands; 2 Department of Experimental Psychology, University of Groningen, Groningen, The Netherlands; 3 BCN-NeuroImaging Center, University Medical Center Groningen, University of Groningen, Groningen, The Netherlands; University of Texas at Dallas, United States of America

## Abstract

While some elderly show deteriorations in cognitive performance, others achieve performance levels comparable to young adults. To examine whether age-related changes in brain activity varied with working memory performance efficiency, we recorded electroencephalography (EEG) from young and older healthy adults during performance on an n-back task with two loads (0- and 1-back) and two versions (identity and integrated). Young adults showed a typical P3 amplitude pattern with a parietal-maximum. Compared to young adults, the P3 amplitude of older adults was characterized by frontal hyperactivity coupled with posterior hypoactivity. Moreover, P3 amplitude in young and older adults varied with working memory performance efficiency. Among young adults, more efficient performance correlated with a larger P3 amplitude at parietal sites. In contrast, a higher P3 amplitude at midline electrode sites in older adults correlated with less efficient performance. Particularly, the enhanced frontal midline EEG activity in older adults during working memory performance seems to reflect inefficient use of neural resources due to frontal lobe dysfunction.

## Introduction

Working memory is defined as a cognitive function with limited capacity that allows individuals to store and actively manipulate information over a brief period of time [1]. With age, working memory becomes less efficient (see [2]). Neuroimaging studies have associated aging with both reduced and increased brain activity. Reduced activity is particularly observed in occipital-temporal regions [3] and seems to reflect cognitive decline [4]. Enhanced activity is generally observed in frontal brain regions [5]–[10].

Until recently, the majority of studies investigating age-related cognitive decline focused on the differences between young and older adults. However, decline in cognitive functions is not a defining feature of all elderly participants. While some older adults show lower performance levels during working memory and related tasks, others achieve levels comparable to their young counterparts [3], [8], [11]. It has been hypothesized that these high performing individuals obtain equivalent performance levels through the functional reorganization of neural networks, in order to compensate for age-related changes in the brain [9], [12]. In a positron emission topography study, Cabeza and colleagues [8], for example, found that young adults and low performing older adults activated similar regions in the right prefrontal cortex (PFC), while high performing older adults activated bilateral PFC regions. The bilateral pattern of frontal activation (known as hemispheric asymmetry reduction in older age or HAROLD [9]), was interpreted as a reflection of the presence of compensatory mechanisms in high performing adults. Differences between high and low performing older adults have also been found in studies using electroencephalography (EEG). Daffner and colleagues [11] observed that compared to low performers, high performers had a larger P3 amplitude with a shorter latency, which increased with task demands. The authors suggested that these high performing individuals employed their cognitive resources more efficiently than low performers. This performance related effect in their study was present in both young and older adults. In contrast, Nagel et al. [13] reported no evidence of compensatory recruitment in working memory related networks; using functional magnetic resonance imaging they found that the brain activation of high performing older adults resembled that of low performing young individuals.

Thus, optimal performance in older adults has been associated with different patterns of brain activity. Some studies found enhanced frontal brain activation to be beneficial to task performance and interpreted this frontal hyperactivity as evidence of compensatory mechanisms in the aging brain [8], [9], [14], [15]. Other studies found frontal hyperactivity not to be predictive of task performance [16], and in some cases even to be related to decreased task performance [5], [17], [18]. Frontal overactivation in these cases was argued to reflect nonspecific or less efficient recruitment of neural resources during task performance [2], [10]. Furthermore, brain activity patterns similar to that of young adults have also been linked to higher task performance [5], [13]. At this moment, it is unclear whether frontal hyperactivity reflects neural compensation, inefficient recruitment of neural resources, or both.

The current study used ERPs to investigate working memory at a neural level. More specifically, we focused on the P3 component, which is thought to reflect neural activity related to attentional and working memory processes [19]. The P3 component is comprised of two subcomponents, one with a maximal scalp distribution over frontal sites and another with a parietal-maximum distribution [20]. Each of the subcomponents represents a distinct function, which is supported by various cortical generators [21], [22]. The frontal P3 subcomponent (referred to as the P3a) has been associated with stimulus novelty processing [17], [23], whereas the parietal P3 subcomponent (referred to as the P3b) has been related to allocation of attentional resources for updating of working memory contents [20], [24], [25]. The P3 component of older adults is characterized by a delayed latency compared to young adults. Moreover, older adults show decreased parietal amplitudes and increased frontal amplitudes, compared to the P3 pattern of young adults [23], [26].

The main focus of the present study is to investigate whether P3 amplitude in older participants varies with working memory performance efficiency. In this experiment, working memory is tested by means of a modified version of the visual n-back task with two loads, 0- and 1-back. In the 1-back load, the target letter changed continuously, requiring constant updating of working memory. While in the 0-back load, the target letter remained the same (the letter ‘x’) and task demands on working memory are limited. Differences in response speed and performance accuracy between the 0- and the 1-back load, in both task versions were used to quantify memory related changes in performance efficiency. A small difference in response speed and accuracy between the two load conditions was indicative of a more efficient working memory performance. In the current study, we expect the P3 amplitude at frontal and parietal sites to vary with performance efficiency, in both young and older adults. More specifically, we will investigate whether more efficient performance in older adults is associated with (bilateral) frontal overactivation or with an activation pattern similar to young adults.

In addition to the load manipulation this n-back task was presented in two versions, an identity and an integrated version. In the identity version, participants had to attend to only a single feature of the stimulus, the identity of the letter. In the integrated version participants attended to a bound representation of two features, namely the identity and location of a letter. Behavioral evidence suggests that the elderly experience difficulties in remembering bound representations of two or more stimulus features [27]. Hence, we expect the elderly to perform better in the identity than in the integrated version.

In summary, the aim of the present study is to determine whether and how brain activity in older adults varies with working memory performance efficiency.

## Materials and Methods

### Participants

Forty healthy young adults (mean age 19.9 years; range 18–26 years; 20 males) and forty-four healthy older adults (mean age 65.8 years; range 60–74 years; 20 males) participated after giving informed consent. All participants were right handed, had normal or corrected to normal vision, had no history of neurological, psychiatric or vascular disease and were not taking any psychotropic or hypertensive medications. The current study was approved by the local ethics committee of the University Medical Center Groningen, the Netherlands.

### Neuropsychological testing

To verify normal cognitive performance, all participants were tested on an extensive neuropsychological battery, consisting of the Mini Mental State Examination (MMSE; [28]), the Hospital Depression Scale (HADS; [29]), visual-motor sequencing (trail-making test A and B), phonemic fluency (words beginning with the letter “S” and “F”), semantic fluency (professions and animals), working memory (digit span test forward and backward and digit symbol coding test), immediate and delayed recall, as well as recognition (15 words test) and a simple reaction time test. During the simple reaction time task, participants were required to press a response button as fast as possible whenever a red dot appeared on the screen. The red dot appeared for 300 ms and inter trial intervals (ITI) varied randomly between 2000 and 6000 ms. In addition, an estimation of the intelligence quotient (IQ) was obtained through the Dutch Adult Reading test, the Dutch version of the National Adult Reading Test (NART; [30]) and the WAIS-matrix reasoning test [31].

### Cognitive task

**Figure 1:**
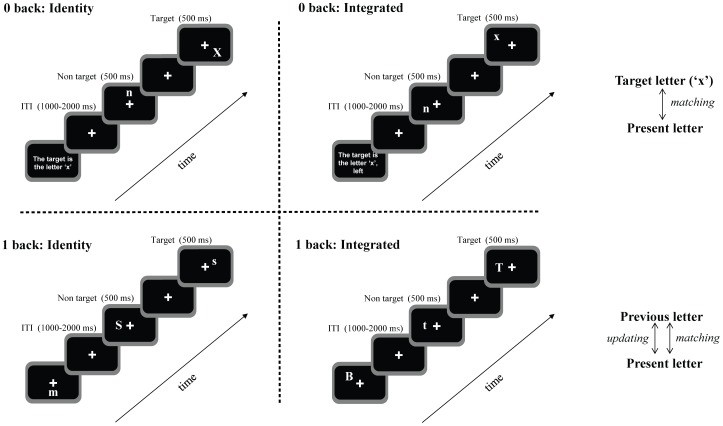
Schematic diagram of the n-back task, separately for each task load, and each task version. Details about timing for stimulus and inter trial interval (ITI) are included.

Working memory was tested using an adapted visual n-back task (Figure 1). Each block started with the presentation of the instructions, followed by a fixation cross, which was presented in the middle of the screen and remained visible throughout stimulus presentation. A continuous stream of letters was presented, one letter per frame, for 500 ms each. The time interval between two stimuli varied randomly between 1000 and 2000 ms. Each letter was randomly positioned in one of 8 possible locations (horizontal×axis, vertical Y axis and the lower and upper position of both diagonals).

Two load conditions of the n-back task were used; 0- and 1-back. In addition, each load had two versions, an identity version and an integrated version. The visual input was identical in all conditions. Participants could differentiate the conditions only through the instructions they received. The use of physically identical stimuli and displays in all conditions avoids the effect of potential confounds of physical stimulus characteristics on performance as well as on brain activity [32].

In the identity version of the 0-back load, the target was the letter ‘x’. In the integrated version of the 0-back load, the target was the letter ‘x’ presented at the left of the fixation cross for half of the participants, or at the right side of the fixation cross for the other half. Hence, in this particular condition, the 2 positions located above and below the fixation cross (vertical axis) were not used, in order to avoid confusion about spatial position.

In the identity version of the 1-back load, the target was any letter identical to the previous letter. In this load, participants were instructed to remember and base their response only on the identity of the presented letter. In the integrated version of the 1-back load, the target was any letter identical to and appearing at the same position as the previous letter. Participants were instructed to memorize and base their response on both the identity and location of the presented letter.

Participants gave their answers manually through a response box. For half of the participants a right index finger response was required for targets and a right middle finger response was required for non-targets. For the other half of the participants index and middle finger responses were reversed.

A total of 4 task blocks (two loads and two task versions) was presented, with 80 trials each. Participants had a break of 1.5 minutes between blocks. In each block, targets occurred randomly in 33–43% of the trials. The order of the task loads and versions was semi-randomized and counterbalanced between participants. These task conditions were part of a larger task set.

Participants were facing the display monitor at a distance of approximately 75 cm; the visual angle between the fixation cross and the letter was approximately 4.3°.The letters were chosen from a set of 18 consonants derived from the Dutch alphabet (all the consonants except the Q, Y and J). Letters were displayed in white in a 40 point Arial font on a black background and were randomly presented either in uppercase (50%) or lowercase (50%). Participants were instructed to ignore the case of the consonant and to focus on its identity (and spatial position). For stimulus presentation a Pentium IV CPU with a 17 inch monitor was used, which had a refresh rate of 100 Hz. E-Prime 1.2 software (Psychology Software Tools Inc., Pittsburgh USA) was used for stimulus generation and response registration.

### EEG recording

EEG was recorded using 64 tin electrodes attached to an electrocap (ElectroCap International Inc., Eaton, Ohio, USA). The electrodes were placed according to the international 10–10 system. The amplifier was a REFA 8–72 (Twente Medical, Systems, Enschede, The Netherlands). An average reference montage was used. Sample frequency was 500 Hz. Two electrodes were placed at the mastoids and were used for off-line re-referencing of the EEG signal. An electrode placed on the sternum served as the participants ground. Four electrodes, placed at the left and right lateral canthi and above and below the left eye, were used to measure the Electro-Oculogram (EOG). Data acquisition was performed using Brain Vision Recorder (version 1.03, BrainProducts GmbH, Munich, Germany).

### Procedure

Participants visited the laboratory twice. During the first visit, demographical and health questionnaires were filled out, neuropsychological tests were administered and the experimental tasks were practiced. Participants trained at least 25 trials for each of the 4 task conditions. Trials were added, until the task was fully clear to the participant. Throughout the practice session, participants received feedback on their performance level after each task condition. In general, the elderly practiced more trials than young adults. During the second visit, participants practiced again after which they performed the n-back task while EEG was recorded. During the EEG recording, participants were seated comfortably in a sound-attenuated, electrically shielded and dimly lit room. Participants were instructed to minimize head and body movements and to keep their eyes fixated on a cross presented in the middle of the screen throughout stimulus presentation. During the task, they were instructed to react as fast and as accurately as possible.

### Performance efficiency

To examine whether performance level was related to specific modulations of brain activity, we determined performance efficiency in young and old adults by taking both accuracy scores and RTs into account. First, individual mean RT and accuracy scores were calculated for the 0- and the 1-back load, by averaging over targets and non-targets, separately for the identity and the integrated version. Second, the difference in RT and accuracy scores between the 1-back load and the 0-back load was calculated for each individual, separately for the identity and the integrated version. A larger difference between the loads (hereafter called the load effect) indicates a more pronounced decrease in performance in the 1-back task that imposes higher demands on the memory system than the 0-back condition. The randomization order in which the two loads were presented (a) first the 0-back followed by the 1-back load and b) first the 1-back followed by the 0-back load did not have differential effects on performance in young and in older adults. Subsequently, the load-effect in RT and accuracy in each version, was standardized (to obtain z scores), for young and older adults separately. After this step, four z-scores were obtained: 1) a z-score representing the load effect in RT in the identity version, 2) a z-score representing the load effect in accuracy in the identity version, 3) a z-score representing the load effect in RT in the integrated version and 4) a z-score representing the load effect in accuracy in the integrated version. For each version, the z-scores representing the load effect in RT and accuracy were averaged for each individual, resulting in a single measure of (working memory) performance efficiency (per version). Note that lower scores in the above-mentioned variables reflect more efficient performance, whereas higher scores indicate less efficient performance.

### Data analysis

#### Behavioral data

Outcome measures included performance accuracy and reaction time (RT), calculated separately for the 0- and 1-back load for the identity and integrated version, as well as, for targets and non-targets. Responses faster than 200 ms and slower than 1500 ms were considered as incorrect.

Data obtained from thirty-seven healthy young adults and thirty-six old adults were considered for further behavioral and ERP analysis. Two young and six older participants were excluded based on their performance level (less than 50% correct responses in one of the conditions). It was concluded that they did not follow the task instruction correctly. Data from one young and two older participants were lost due to technical problems.

#### ERP data

ERP data was processed using Brain Vision Analyzer 2 software (Brain Products, Munich, Germany). The EEG signal was filtered with a Butterworth high-pass filter of 0.16 Hz (48 dB/oct) and a low-pass filter of 30 Hz (48 dB/oct). Only correct trials were included for further analysis. The algorithm of Gratton, Coles and Donchin [33] was used to correct ocular movements artifacts. Further artifact removal was applied by removing segments with an absolute difference larger than 200 µV or a voltage step per sampling point larger than 50 µV. Baseline correction was applied from -200 ms until stimulus onset. Epochs were averaged starting 200 ms before stimulus onset and lasting until 1000 ms post-stimulus onset, separately for each load, version and stimulus type. P3 peak detection was performed on individual ERP data at F5, Fz, F6, C5, Cz, C6, P5, Pz and P6, using a semi-automatic peak detection method (using Brain Vision Analyzer, v. 2.0), in a window from 300 ms to 700 ms post-stimulus. Peak detection was performed separately at each electrode and for each task condition. The average amplitude in a symmetric 50 ms interval around peak latency was calculated. Moreover, to examine age-related differences in early perceptual stages, N1 peak detection was performed on individual ERP data at PO7 and PO8, using a semi-automatic peak detection method, in a window from 120 ms to 220 ms post-stimulus. For the N1 component, the amplitude at peak latency was used in the analysis.

#### Statistical analysis

Behavioral and ERP data were analyzed by means of linear mixed effect models, using the lmer and pvals.fnc functions in the lme4 library [34] as well as the pamer.fnc function in the “LMERConvenienceFunction” library [35] running in R (see [36]–[38]). Accuracy scores were modeled by a binominal mixed effect model, using a logit-link function fitted by a Laplace approximation of the likelihood function. The categorical factors age (young and old), load (0- and 1-back), version (identity and integrated) and stimulus type (target and non-target) were effect-coded (−1,1) and then entered as fixed factors. The factor age was also centered before entering into the model. In addition, ‘subject’ was entered as a random factor. The estimated parameters and z-statistics of the binominal model are reported.

RTs (per trial) as well as ERP latencies and amplitudes were modeled by separate non-binominal models, with the same effect-coded factors as for the accuracy scores. Before entering the model, RTs and latencies were converted to seconds. For the analysis of N1 data, two additional fixed factors were entered, the effect-coded factor electrode (PO7 and PO8) and the continuous factor performance efficiency. The analysis of the P3 data included three additional fixed factors, lateralization (left, midline, right), electrode (frontal, central, parietal) and performance efficiency. The single factor performance efficiency contained performance efficiency in the identity as well as performance efficiency in the integrated version. This factor was implemented in such a way that performance efficiency in the identity version was associated with latencies and amplitudes in the identity version. Likewise, performance efficiency in the integrated version was associated with ERP data in the integrated version.

For all models, a (backward) stepwise model selection was implemented to choose the best fitting ‘reduced’ statistical model. For the (backward) stepwise model selection we followed the procedure described by Newton and colleagues [35]. The back fitting of the fixed effects predictors was performed with the “LMERConvenienceFunction” in R, a function based on log-likelihood ratio testing. For the analysis of both P3 latency and amplitude data, the complex model (containing all main effects and interactions) was entered first. Subsequently, simpler models containing fewer interactions between the predictors were generated in a consecutive manner and compared with the previous complex model until the best fitting model was identified. The best fitting model contained a reduced number of interactions between predictors than the complex model. The interactions between factors that did not account for significant variance were removed from the analysis [38]. For complex statistical models, containing factors with 3 levels or more (such as the analysis of the P3 data), main effects and interactions between the predictors were investigated with the ‘pamer.fnc’ function, comparable to an ANOVA F-test.

For the non-binominal models of RT and ERP data, the p-values were calculated using Markov Chain Monte Carlo (MCMC) sampling, with 10000 samples. The upper and lower highest posterior density (HPD95) [38] intervals are reported. Comparable to the traditional 95% confidence intervals, the HPDs reflect the minimum and maximum value of the expected range of the estimated parameters. Significant age-and performance-related effects related to task load and task version are described in the results section. Interaction effects that were not supported by significant post-hoc tests are not reported.

Finally, neuropsychological test scores were analyzed by means of separate one-way ANOVAs, including performance efficiency in the identity and in the integrated version as covariates of interest. Interactions between neuropsychological test scores and performance efficiency in each version were assessed by Spearmans's rank correlation coefficient. Statistical significance was set at α<0.05.

## Results

### Neuropsychological testing

Main demographics and neuropsychological test results are presented in Table 1. Young and older adults did not differ in average WAIS matrix IQ scores. However, older adults had lower average MMSE scores (F(1,67) = 12.8, p = 001) and higher crystallized intelligence scores (as measured by the NLV IQ score F(1,67) = 15.1, p<0005) than young participants. Moreover, older adults were slower in the visual-motor sequencing task (F(1,67) = 29.2, p<0005 and F(1,67) = 27.8, p<0005 for trail making A and B, respectively) and had lower scores on the following measures of memory: WAIS digit span backwards (F(1,67) = 14.5, p<0005), the 15-words test comprising of direct recall (F(1,66) = 93.7, p<0005), delayed recall (F(1,66) = 63.5, p<0005) and recognition (F(1,66) = 22.6, p<0005). Older adults were slower in a simple reaction time test than young adults (F(1,67) = 7.7, p = 007).

**Table 1:** Demographics and neuropsychological test results.

	Young			Old		
		Mean	SD		Mean	SD
Total number		37			36	
Gender, male/female		18/19			16/20	
Age, years ^1^		19.7	1.6		65.6	3.7
MMSE ^1^		29.4	0.7		28.7	1
NLV IQ ^1^		101.7	5.2		109.3	10.4
WAIS matrix IQ		111.5	9		108.8	10.9
Visual-motor sequencing						
Trail making A ^1^		25.4	7.3		38.6	12.6
Trail making B ^1^		53.6	13.2		79	26.6
Memory tasks						
WAIS number Forwards		10.1	2.1		9.1	2.2
WAIS number Backwards ^1^		7.8	2.1		6	1.8
*15 words test Direct recall ^1^		57.8	6.6		40.3	9.1
*15 words test Delayed recall ^1^		12.3	2.1		7.6	3
*15 words test Recognition ^1^		29.8	0.4		28.1	2.1
Simple reaction time task ^1^		218.7	20.4		233.8	24.8

^1^ Old and young adults differ significantly; *One old adults did not perform the 15 words test; MMSE = Mini Mental State Examination, NLV = Dutch Adult Reading test, WAIS matrix = matrix reasoning subtest of the Wechsler Adult Intelligence Scale.

Older adults with less efficient working memory performance in the integrated version were slower in the Trail making B test (r = 339, p = 043), whereas performance efficiency in the integrated version in young adults was not associated with Trail B test scores (F(1,67) = 4.2, p = 043). Moreover, young, but not older, adults with a more efficient performance in the integrated version had higher scores in the direct recall subtest of the 15 words test (−360, p = 029; (F(1,66) = 6.6, p = 012).

### Behavioral data

**Figure 2:**
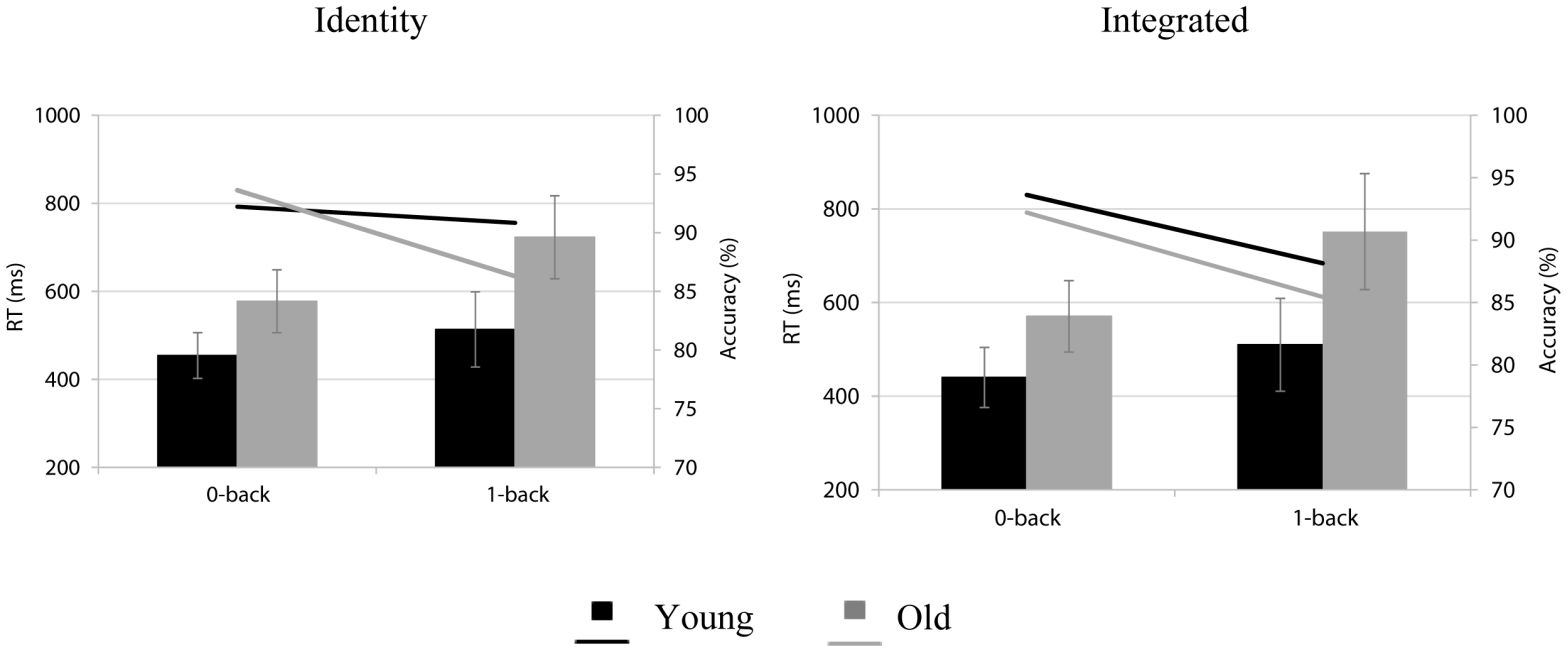
Behavioral results comprised of mean RTs (bars) in milliseconds (ms) and accuracy scores (lines) in percentages (%) for young (black) and old (grey) adults. Behavioral outcomes are presented, separately, for the 0-back and the 1-back load in the identity version and in the integrated version.

Mean accuracy scores and RTs for young and older adults, for the different task conditions are illustrated in Figure 2. The estimated proportion of correct responses was higher in the 0-back (92%) than in the 1-back load condition (90%; β_intercept_  = 2.33; z  = 34, p<0.0005; β_load_  = −.35; z  = −7.7, p<0.0001). However, this load effect was modulated by age and version (β_age*load*version_  = −.62; z  = −3.4, p<0.001). For older adults, the estimated proportion of correct responses was higher in the 0-back than in the 1-back load condition, in both versions (identity: 92.6% vs. 87.6%; β_intercept identity version in the 0-back load_  = 2.54 z  = 20.1, p<0.0001; β_1-back_  = −.55; z  = −6.14, p<0.0001 and integrated: 92.4% vs. 88.5%; β_intercept integrated version in the 0-back load_  = 2.49, z  = 17.91, p<0.0001; β_1-back_  = −46; z  = −5.25; p<0.0001). For young adults, this load effect was limited to the integrated version (93.6% vs. 90%; β_intercept integrated version in the 0-back load_  = 2.66, z  = 24.22, p<0.0001; β_1-back_  = −.47; z  = −4.98, p<0.0001).

In general, older adults reacted slower (680 ms) than young participants (488 ms; β_intercept = _583, HPD95 = 569, 599, p = 0.001; β_age_ = 192, HPD95 = 164, 223, p = 0.001). All participants were slower in the 1-back than in the 0-back load condition (β_load_ = 120, HPD95 = 116, 125, p = 0.001). However, this load effect was more pronounced in older adults (mean difference between load conditions 164 ms) than in young adults (74 ms; β_age*load_ = 087, HPD95 = 078, 095, p = 0.001). An interaction between age, load and version (β_age*load*version_  = −024, HPD95  = −041, −008, p = 0.003) revealed that in the 1-back load condition, older adults were slower in the integrated (778 ms) than in the identity version (748 ms; β_intercept integrated version in the 1-back = _780, HPD95 = 749, 809, p = 0.0001; β_identity version in the 1-back_  = −029, HPD95  = −040, −018, p = 0.0001), whereas in the 0-back load condition they had similar RTs for both versions. Young adults showed no version-dependent differences RT in any of the load conditions.

### N1 component

There were no significant age- and/or performance efficiency related effects on N1 latency and amplitude, with regard to task load and/or task version.

### P3 component

#### P3 latency

Old adults had a later P3 latency than young adults, at all parietal electrodes and at Fz (age*lateralization*electrode: F(4,5130) = 13.3, p<0001; see Table 2).

**Table 2:** Mixed-effects model for age-related effects on P3 latency, related to electrode site.

Intercept: mean latencies (seconds) for young adults				β _older adults_	
	Intercept (young)	Estimate of difference between young and old	HPD95 lower	HPD95 upper	pMCMC
**Fz**	.40	.02	.01	.38	.0002
**F5**	.47	−.03	−.06	.01	.097
**F6**	.47	−.02	−.06	.01	.012
**Cz**	.41	.02	−.00(1)	.03	.076
**C5**	.44	.03	.00(02)	.06	.052
**C6**	.44	.02	−.01	.04	.206
**Pz**	.40	.03	.02	.05	.0001
**P5**	.40	.07	.05	.09	.0001
**P6**	.39	.06	.04	.08	.0001

The P3 latencies were converted in seconds prior to being fitted in the model.

Furthermore, age-related differences in P3 latencies were modulated by task load and version (age*load*version: F(1,5130) = 6.1, p = 014). Young adults had longer latencies in the identity compared to the integrated version, in both load conditions (0-back: 427 ms vs. 414 ms; β_intercept for young adults in the identity version in the 0-back = _429, HPD95 = 413, 445, p = 0.0001; β_integrated_ _version in the 0-back_  = −011, HPD95  = −019, −002, p = 018 and 1-back: (430 ms vs. 412 ms; β_intercept for young adults in the identity version in the 1-back = _434, HPD95 = 418, 451, p = 0.0001; β_integrated_ _version in the 1-back_  = −015, HPD95  = −024, −.005, p = 004). For the elderly, this effect was limited to the 0-back load condition (450 ms vs. 430 ms; β_intercept for older adults in the identity version in the 0-back = _454, HPD95 = 440, 468, p = 0.0001; β_integrated_ _version_  = −.011, HPD95  = −.019, −.003, p = 007).

An interaction between age group, task version and performance efficiency was observed (F(1,5130) = 19, p<0001). For the elderly, slower P3 latencies in the identity version were associated with less efficient performance in this version (β_performance identity in older adults = _005, HPD95 = 001, 008, p = 013). In the integrated version, performance efficiency and P3 latencies in the elderly were not significantly related to each other. In young adults, performance efficiency in the identity or in the integrated version was not related to P3 latency in the respective versions.

#### P3 amplitude

**Figure 3:**
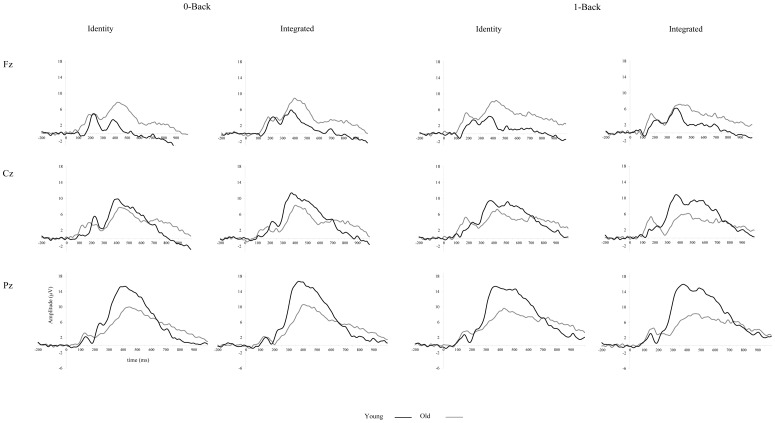
Grand average ERP plots for the midline electrodes for targets in the 0- and 1-back load, separately for the identity and integrated version, for young (black lines) and for old (gray lines) adults. Fz, Cz and Pz are presented from top to bottom.

Young adults had a typical P3 amplitude pattern, with a parietal-maximum. Compared to young adults, the P3 amplitude of older participants was higher at frontal sites and lower at posterior electrodes, indicating an anterior-shift of the P3 (age*electrode: F(4,5108) = 474.4, p<0001; see Figure 3). Lateralization effects in both young and older adults were subtle and depended on electrode site (see Table 3 for specific details; age*electrode*lateralization: F(4,5108) = 10.7, p<0001).

**Table 3:** Mixed-effect model estimates for age-related effects on P3 amplitude, related to electrode site.

Intercept: mean amplitude ( μV) for young adults				β _older adults_	
	Intercept (young)	Estimate of difference between young and old	HPD95 lower	HPD95 upper	pMCMC
**Fz**	3.53	3.05	2.00	4.14	.0001
**F5**	3.48	2.94	1.72	4.14	.0001
**F6**	5.16	2.48	1.22	3.67	.0001
**Cz**	8.60	−2.66	−3.92	−1.44	.0001
**C5**	5.73	.10	−.79	1.02	.827
**C6**	7.58	−.86	−1.84	.18	.098
**Pz**	13.85	−5.37	−6.70	−4.16	.0001
**P5**	10.52	−3.70	−4.67	−2.74	.0001
**P6**	10.83	−3.19	−4.26	−2.14	.0001

Performance efficiency had differential effects on P3 amplitude in young and in older adults, depending on lateralization (age*lateralization*performance: F(2,5108) = 9.4, p<0001) and electrode site (age*electrode*performance: F(2,5108) = 12.8, p<0001). These effects were not dependent on task version, indicating that performance efficiency in the identity and in the integrated version showed a similar relation to P3 amplitude in the respective versions. Among older adults, higher P3 amplitude at midline sites was associated with less efficient performance (β_performance efficiency for older adults = _158, HPD95 = 006, 333, p = 038). For young adults, the relationship between performance efficiency and P3 amplitude was not dependent on lateralization. In contrast, among young adults, efficient performance was associated with higher amplitude at parietal electrodes (β_performance efficiency for young adults_  = −.352, HPD95  = −.599, −.157, p = 001. For older adults, the relationship between performance efficiency and P3 amplitude in either task version was not dependent on electrode site

Young adults had a larger P3 amplitude in the 1-back load condition (8 µV) compared to the 0-back load condition (7 µV; β_intercept for young adults in the 0-back_  = 7.437, HPD95  = 6.551, 8.344, p = 0.0001; β_1-back = _521, HPD95 = 156, 890, p = 006), whereas older adults had comparable P3 amplitudes for both load conditions; age*load: F(1,5108) = 6, p = 014).

Furthermore, there was an interaction between age, load and version (F(1,5108) = 5.1, p = 025). Young adults showed higher P3 amplitudes in the integrated version than in the identity version, for both load conditions (0-back: identity 7 µV vs. integrated 8 µV; β_intercept for young adults in the identity version in the 0-back_  = 6.816, HPD95  = 5.843, 7.720, p = 0.0001; β_integrated_ _version in the 0-back_  = 1.243, HPD95 = 719, 1.738, p = 0001 and 1-back: identity 7 µV vs. integrated 9 µV; β_intercept for young adults in the identity version in the 1-back_  = 7.435, HPD95  = 6.462, 8.393, p = 0.0001; β_integrated_ _version in the 1-back_  = 1.044, HPD95 = 502, 1.557, p = 0002). For the elderly, this effect was limited to the 0-back load condition (identity 6 µV vs. integrated 7 µV; β_intercept for older adults in the identity version in the 0-back = _6.484, HPD95  = 5.7403, 7.273, p = 0.0001; β_integrated_ _version in the 0-back = _757, HPD95 = 398, 1.111, p = 0001)

In addition, an interaction between age, load, version and performance efficiency was present (F(1,5108) = 3.8, p = 023). For both age groups in the identity version, performance efficiency was not significantly associated with P3 amplitude in any of the loads. For young adults, in the integrated version, efficient performance was associated with larger amplitude in both load conditions, particularly in the 1-back (0-back: β_performance efficiency integrated version_  = −.652, HPD95  = −1.274, −.060, p = 037 and 1-back: β_performance efficiency integrated version_  = −.762, HPD95  = −1.361, −.143, p = 015). This effect was reversed in the elderly in the integrated version, that is, less efficient performance was associated with higher amplitude in both load conditions, particularly in the 1-back load condition (0-back: β_performance efficiency integrated version = _584, HPD95 = 113, 1.069, p = 017 and 1-back: β_performance efficiency integrated version = _719, HPD95 = 246, 1.195, p = 003).

## Discussion

The current study investigated the association between performance efficiency and ERPs in young and older adults, during a working memory task with two loads (0- and 1-back) and two versions (identity and integrated). In the current task, working memory performance efficiency is reflected in an increase in RTs and decrease in accuracy scores from the 0- to the 1-back load condition. In the 1-back load condition, the target letter changed continuously, requiring constant updating of working memory, while in the 0-back load condition, the target letter remained the same (the letter ‘x’). The increase in response times and decrease in accuracy scores with increasing task load was more pronounced in older than in young adults. Conform previous findings, higher P3 amplitudes in young adults were associated with better task performance [19], particularly at parietal electrodes. For the elderly, the opposite pattern was observed. That is, for these participants a lower P3 amplitude at midline electrodes was associated with a more efficient performance. These effects could not be explained by age-related changes in early perceptual processes, as the N1 latency and amplitude were similar in the young and older adults and did not show a relation with performance efficiency.

P3 amplitude has been shown to increase with the amount of attentional demands required by the task at hand [19], [25], [39]. Therefore, it has been suggested that P3 amplitude reflects the engagement of attention in updating the contents of working memory [19], [20], [24], [25]. Young adults in the current study showed a typical P3 amplitude pattern with a parietal maximum [20], [40]. In the 1-back load, young adults had a higher P3 amplitude and decreased behavioral performance compared to the 0-back load, suggesting that attentional demands were higher in the 1-back load condition. Therefore, the positive association between higher P3 amplitude and better performance found in young adults, seems to reflect that high performing individuals achieve better performance by paying more attention to the task.

Compared to young adults, older adults had slower P3 latencies and lower P3 amplitude at parietal sites. The delayed parietal P3 with a decreased amplitude in older adults has been interpreted as evidence of cognitive decline [41]–[43] due to neural decrements in posterior brain regions. Consistent with this cognitive decline account, older adults in this study performed less efficiently than young adults. If aging indeed impairs neural generators projecting to posterior brain sites, then a lower P3 amplitude is expected to correlate with less efficient task performance in older adults. Contrary to these expectations, more efficient performance in our old adults was associated with lower midline parietal P3 amplitude. Our findings are in line with the results of Gajewski and colleagues [44], who recorded EEG during performance on a task-switching paradigm involving working memory processes. Their results showed that healthy elderly carriers of the ‘Met-allele’ performed better and had lower parietal P3 amplitude than those elderly who did not carry this specific genetic marker.

Other studies have also found decreased posterior activation to be associated with optimal performance, but only when it was additionally accompanied by increased (bilateral) frontal activation [3], [6], [8], [45], [46]. This additional frontal activation in old adults has been argued to serve a compensatory function, used to counteract age-related neuronal decline in posterior brain regions [4], [47].

In the current study, older adults showed a more anterior-oriented P3 pattern compared to young adults [22], [48]. However, amplitude lateralization was similar in both age groups. The higher P3 amplitude at the frontal midline electrode in our older adults was associated with less efficient performance. This effect was similar to the pattern observed on the parietal P3. Taken together, it is unlikely that the enhanced frontal activation observed in our older adults reflects (successful) neural compensation.

Alternatively, the more frontally oriented P3 component in older adults may reflect inefficient recruitment of frontal brain resources [5], [17], [18], [48]. Among others, Fabiani and colleagues [5] found that older participants with a frontal-maximum P3 scalp distribution pattern performed poorly on an oddball task. In contrast, older adults with a parietal-maximum P3 topography, similar to young adults, were found to perform better during this task. The authors argued that in low performing older adults frontal brain areas become dysfunctional and in order to compensate for this decline, they rely on additional recruitment of these frontal regions. This frontal hyperactivity has been related to working memory processes in particular, reflecting (unsuccessful) compensation for faster decay of memory traces, lower working memory capacity, and/or inefficient suppression of irrelevant information [5], [48]. Additional support for the inefficient employment of frontal brain areas in our older adults is found in the relationship between less efficient performance in the integrated version and the longer “completion time” in the Trail making B test, a neuropsychological test used to measure executive functions [49], which are supported by the frontal cortex [50].

Despite the better performance achieved in the easy (0-back) than in the more demanding condition (1-back), it should be noted that older adults had similar P3 amplitude in both load conditions. These results suggest that the decline in age-related frontal lobe functions results in a maximal recruitment of neural resources already during the easy task load condition. Ceiling effects might have prevented a further increase in activity thus explaining the lack of load dependent modulation of the P3 amplitude in older adults. Performance efficiency in older, but also young, adults was associated with P3 amplitude in both load conditions. This suggests that the “amplitude - performance’ association is driven by more general task components that are present in both load conditions, and is less related to specific working memory functions (see [51], for a similar interpretation regarding memory load).

The relationship between performance efficiency and P3 amplitude seems to be partially in conflict with the findings of Daffner et al [11] who linked a higher performance level to a larger parietal and frontal P3 amplitude increase with increasing task load. The authors based performance level of their participants on accuracy scores observed in the 2-back load condition. It is known that, compared to the 1-back load condition, the 2-back load condition is more demanding and requires additional working memory processing related to updating of the temporal order of stimuli. In line with our findings, the P3 amplitude pattern observed in the 0- and the 1-back load condition seems to be higher for the low performing than the high performing older adults in the data reported by Daffner et al [11] (see their discussion section as well as Figures 4, 7, and 8).

Modulations of P3 amplitude related to task version were observed, as well. The association between P3 amplitude and performance efficiency was most pronounced in the more complex condition, that is, the integrated version. Young adults had larger P3 amplitude in the integrated than in the identity version. Elderly, however, showed a smaller difference in P3 amplitude between the identity version and integrated version, which was limited to the 0-back load. Moreover, they were as accurate in both versions, although differences in response time were observed in the 1-back load condition. In general, these findings do not offer strong support to previous studies reporting age-related impaired performance during conditions in which two or more features needed to be integrated in a bound representation [27].

In summary, the present study investigated how normal aging modulates ERP latency and amplitude during performance on a working memory task and how these modulations were associated with performance efficiency. In general, older adults performed less efficiently than young adults. Moreover, P3 amplitude in the elderly participants was found to be lower at parietal sites and higher at frontal sites, compared to young adults. The relation between performance efficiency and P3 amplitude was found to be dependent on age and scalp location. Higher parietal P3 amplitude in young adults correlated with more efficient performance, whereas in the elderly, a higher P3 amplitude at midline sites correlated with less efficient performance. Particularly, the enhanced frontal midline EEG activity in old adults during working memory performance seems to reflect inefficient use of neural resources, due to frontal lobe dysfunction.
